# Pain Intensity and Unpleasantness in People with Vascular Dementia: A Cross Sectional Study

**DOI:** 10.1093/geroni/igaa057.3249

**Published:** 2020-12-16

**Authors:** Angela Humbel, R Cowan, Mary Dietrich, Wm Larkin Iversen, Sebastian Atalla, Karen Moss, Sydney Englehart, Todd Monroe

**Affiliations:** 1 The Ohio State University, Columbus, Ohio, United States; 2 The University of Tennessee Health Science Center, Memphis, Tennessee, United States; 3 Vanderbilt University, Nashville, Tennessee, United States

## Abstract

Pain is a multidimensional sensory and affective experience. People with Vascular Dementia (VaD) experience pain more intensely and have negative emotional responses. Further investigation is needed to understand the neurobiology of pain in VaD. We used experimental thermal pain in a cross-sectional design to determine if adults (age>64) with probable VaD experience increased pain intensity and increased pain unpleasantness during “mild” and “moderate” thermal pain. The final sample included 46 sex- and age-matched adults (23 VaD; 12 female) and controls (23 cognitively intact; 12 female) with an average age of 76.5 years (SD=7.5). Participants reported no daily analgesic use. We used a thermode placed on the thenar eminence to assess temperatures perceived as mild and moderate pain (°C) followed by unpleasantness ratings (0-20 scale). We assessed cognition and depression with the Mini-Mental State Exam (MMSE) and the Geriatric Depression Scale. After controlling for depression, and relative to controls, there was no statistically significant difference in the temperature at which people with VaD perceived mild or moderate pain (p = .086; Cohen’s d: mild=0.55, moderate=0.27). However, there was a statistically significant effect of VaD status on pain unpleasantness (p = .003). People with VaD reported mild and moderate pain as more unpleasant than controls (Cohen’s d = 0.79 and 0.60, respectively). Findings support previous work that people with VaD are at risk of experiencing more pain. Assessing pain intensity and affect can avoid under-treated pain in those with VaD.

